# Formyl Peptide Receptor 2-Dependent cPLA2 and 5-LOX Activation Requires a Functional NADPH Oxidase

**DOI:** 10.3390/antiox13020220

**Published:** 2024-02-08

**Authors:** Tiziana Pecchillo Cimmino, Iolanda Panico, Simona Scarano, Mariano Stornaiuolo, Gabriella Esposito, Rosario Ammendola, Fabio Cattaneo

**Affiliations:** 1 Department of Molecular Medicine and Medical Biotechnology, School of Medicine, University of Naples Federico II, 80131 Naples, Italy; tiziana.pecchillocimmino@unina.it (T.P.C.); io.panico@studenti.unina.it (I.P.); simon.scarano@studenti.unina.it (S.S.); gabriella.esposito@unina.it (G.E.); rosario.ammendola@unina.it (R.A.); 2 Department of Pharmacy, School of Medicine, University of Naples Federico II, 80131 Naples, Italy; mariano.stornaiuolo@unina.it

**Keywords:** arachidonic acid, cell metabolism, Formyl Peptide Receptor, FPR2, NADPH oxidase, ROS, 5-LOX, cPLA2, redox pathway

## Abstract

Phospholipases (PL) A_2_ catalyzes the hydrolysis of membrane phospholipids and mostly generates arachidonic acid (AA). The enzyme 5-lipoxygenase (5-LOX) can metabolize AA to obtain inflammatory leukotrienes, whose biosynthesis highly depends on cPLA2 and 5-LOX activities. Formyl Peptide Receptor 2 (FPR2) belongs to a subfamily of class A GPCRs and is considered the most versatile FPRs isoform. Signaling triggered by FPR2 includes the activation of several downstream kinases and NADPH oxidase (NOX)-dependent ROS generation. In a metabolomic analysis we observed a significant increase in AA concentration in FPR2-stimulated lung cancer cell line CaLu-6. We analyzed cPLA2 phosphorylation and observed a time-dependent increase in cPLA2 Ser505 phosphorylation in FPR2-stimulated cells, which was prevented by the MEK inhibitor (PD098059) and the p38MAPK inhibitor (SB203580) and by blocking NOX function. Similarly, we demonstrated that phosphorylation of 5-LOX at Ser271 and Ser663 residues requires FPR2-dependent p38MAPK and ERKs activation. Moreover, we showed that 5-LOX Ser271 phosphorylation depends on a functional NOX expression. Our overall data demonstrate for the first time that FPR2-induced ERK- and p38MAPK-dependent phosphorylation/activation of cPLA2 and 5-LOX requires a functional NADPH oxidase. These findings represent an important step towards future novel therapeutic possibilities aimed at resolving the inflammatory processes underlying many human diseases.

## 1. Introduction

Arachidonic acid (AA) is a ω-6 polyunsaturated fatty acid present in membrane phospholipids that participates in many cellular physiological processes, including inflammation and immunity [1]. Phospholipases (PL) (EC 3.1) are hydrolase enzymes that share the ability to catalyze the hydrolysis of membrane phospholipids [2] and are classified based on the site cleaved in the phospholipid molecule [3]. The hydrolytic cleavage catalyzed by PLA2 generates polyunsaturated fatty acids, mostly AA and lysophospholipids, thus determining the phospholipid composition of membranes [4]. The PLA2 (PLA2 EC 3.1.1.4) superfamily includes cytosolic (cPLA2), secreted (sPLA2), calcium-independent (iPLA2), platelet activator acetyl-hydrolase (PAF-AH), lysosomal (LPLA2), and adipose tissue-specific PLA2s (AdPLA) phospholipase. The first two classes of these enzymes are highly expressed in tumor cells [5].

Eicosanoids are a family of bioactive compounds derived from AA that play crucial roles in pathophysiology, including inflammatory conditions of multiple organ systems. They include prostaglandins (PG), thromboxanes (TX), leukotrienes (LT), and lipoxins (LX). Once released by the catalytic action of cPLA2, AA can be metabolized by cyclooxygenases (COXs) that catalyze the transformation of AA in PGH2 [6,7]. The aberrant AA metabolism observed in cancer cells results in a high concentration of PGs, in particular PGE_2_ [8,9]. Alternatively, AA can be metabolized by the enzyme 5-lipoxygenase (5-LOX) to generate LTs. 5-LOX interacts with 5-lipoxygenase activating protein (FLAP) and converts AA to LTA4 [10]. Therefore, LTs biosynthesis is highly dependent on the activities of cPLA2 and 5-LOX. In addition, AA can be metabolized by cytochrome P450, generating epoxides and a wide spectrum of biologically active fatty acid mediators [9].

The biosynthesis of eicosanoids requires several catalytic steps regulated by inflammatory and stress signals via different molecular mechanisms. In fact, phosphorylation at Ser505 residue of cPLA2 by extracellular response kinases (ERKs) and the stress-regulated p38MAPK regulates the activity of this enzyme [11,12], whereas 5-LOX is phosphorylated at Ser271 and Ser663 residues by p38MAPK-regulated MAPKAPK-2/3, ERKs, and CaMKII. Conversely, phosphorylation at Ser523 residue by PKA suppresses 5-LOX activity [13]. The G-protein coupled receptors (GPCRs) can sense extracellular metabolites and thus regulate inflammatory responses, including eicosanoid production [10]. Signaling triggered by GPCRs includes calcium mobilization and the activation of several downstream kinases, such as ERKs, p38MAPK, and p38MAPK-regulated MAPKAPK, which in turn can regulate cPLA2 and 5-LOX activity.

The Formyl Peptide Receptors (FPRs) are a subfamily of class A GPCRs that, in humans, includes three different isoforms (FPR1, FPR2, and FPR3) [14]. The distinct members of this family are functionally expressed on the cellular and nuclear membrane of several cell types [15,16,17]. FPRs can recognize a plethora of ligands, which include both pathogen-associated molecular patterns (PAMPs) and damage-associated molecular pattern (DAMPs) [18,19], modulating several biological functions, such as angiogenesis, metabolism, cell proliferation, and cell death [14,20,21,22]. FPRs are also able to modulate inflammatory responses in many physio-pathological processes, such as cancer [16,23,24,25,26], neurodegeneration [27,28,29], and cardiovascular diseases [30,31].

FPR2 is considered the most versatile FPR isoform, being able to recognize an array of structurally and chemically unrelated ligands [32,33]. FPR2 agonists include both endogenous ligands and exogenous ligands [32]. Depending on the nature of its ligands and/or FPR2 rearrangement with other FPR isoforms or with the scavenger macrophage receptor with collagenous structure (MARCO) [34], FPR2 can modulate pro- or anti-inflammatory responses [14,35].

Intracellular cascades triggered by FPR2 include the activation of several kinases [36,37,38] and of signaling and non-signaling proteins [14,22], as well as the NADPH oxidase (NOX)-dependent release of reactive oxygen species (ROS) [15,39,40]. Notably, low levels of ROS can act as second messengers, and FPR-mediated NOX-dependent ROS generation is crucially involved in transactivation of various Tyrosine Kinase Receptors (RTKs) [41], such as EGFR [38], TrkA [42], c-Met [43], VEGFR [20], and IGFR [44].

We recently demonstrated that the FPR2 agonists WKYMVm and ANXA1 elicit intracellular redox signaling pathways involved in glucose uptake and aerobic metabolism of glucose typical of the Warburg effect in the human lung adenocarcinoma cell line CaLu-6 [44,45]. Moreover, we proved that exposure to FPR2 agonists enhances the non-oxidative phase of pentose phosphate pathway (PPP), improves the expression of the glutamine transporter ASCT2, and induces the de novo synthesis of pyrimidine nucleotides [46].

We herein report our metabolomic data showing a significant increase in AA in FPR2-stimulated CaLu-6 cells. Therefore, we dissect the molecular mechanisms involved in FPR2-dependent cPLA2 and 5-LOX activation that trigger AA release and LTs synthesis, respectively. We also evaluated the role of redox signaling in the cell metabolism of AA, disclosing that FPR2-mediated NADPH oxidase-dependent ROS generation plays a key role in both cPLA2 and 5-LOX phosphorylation and, in turn, in AA metabolism.

## 2. Materials and Methods

### 2.1. Cell Culture and Reagents

Epithelial anaplastic human lung cancer CaLu-6 and p22phox^Crispr/Cas9^ CaLu-6 cells were cultured in Dulbecco’s modified Eagle’s medium (DMEM) supplied with 10% fetal bovine serum (FBS) (Invitrogen Corp., Carlsbad, CA, USA) at 37 °C and 5% CO_2_. Cells at 70% of confluence were serum deprived for 24 h and stimulated or not with 10 μM WKYMVm (Primm, Milan, Italy). Unstimulated CaLu-6 cells were used as negative control. In other experiments, CaLu-6 cells were preincubated with the selective FPR2 antagonist WRWWWW (WRW4) (Primm, Milan, Italy) for 15 min at a final concentration of 10 μM, or with 100 μM apocynin, the selective inhibitor of NADPH oxidase (Sigma Chemical, St. Louis, MO, USA), for 2 h, or with 50 μM PD09805, a selective inhibitor of MEK (Sigma), for 90 min, or with 10 μM SB203580, a selective inhibitor of p38MAPK (Sigma), for 1 h. FPR2-unstimulated CaLu-6 cells only preincubated with the appropriate amount of the above-mentioned selective inhibitor were used as a negative control of pretreatments.

### 2.2. p22phox^Crispr/Cas9^ Double-Nickase CaLu-6 Cells

p22phox^Crispr/Cas9^ cells were generated by transfecting CaLu-6 cells with Double Nickase Plasmid or with a Double Nickase Plasmid control (Santa Cruz Biotechnology, Irvine, CA, USA) following the manufacturer’s instructions, as previously described. The expression of p22phox was analyzed by Western blotting [40].

### 2.3. Metabolomic Analysis by LC-MS

Metabolomic analysis by LC-MS was performed in growing and in 24 h serum-starved CaLu-6 cells stimulated or not with WKYMVm in the presence or absence of WRW4 as previously described [46]. Briefly, 24 h serum-starved cells were treated as above mentioned and lysed in 400 μL of a 1:1 prechilled MetOH:H_2_O solution. The samples were centrifuged at 10,000× *g* at 4 °C for 10 min. Supernatants were dried and then reconstituted with 125 μL of methanol/acetonitrile/water (50:25:25). Extracted metabolites were analyzed using an ACQUITY UPLC system online coupled to a Synapt G2-Si QTOF-MS (Waters Corporation, Milford, MA, USA) in positive and negative modes in the following settings: reverse-phase ACQUITY UPLC CSH C18 (1.7 μm, 100 × 2.1 mm^2^) column (Waters), 0.3 mL/min flow rate, mobile phases composed of acetonitrile/H_2_O (60:40) containing 0.1% formic acid and 10 mM ammonium formate (Phase A), and isopropanol/acetonitrile (90:10) containing 0.1% formic acid and 10 mM ammonium formate (Phase B). Peak detection, metabolite identification, and quantitation were performed as previously described [44], fitting experimental data with internal standard and calibration curves. Data analysis and heatmap were generated with the online software MetaboAnalyst 5.0 (https://www.metaboanalyst.ca, accessed on 1 June 2021).

### 2.4. Protein Extraction and Western Blot

Whole lysates were obtained as previously described [47]. Briefly, 24 h serum-starved CaLu-6 or p22phox^Crispr/Cas9^ CaLu-6 cells were stimulated or not with 10 μM WKYMVm, in the presence or absence of the above-mentioned selective inhibitors, and lysed with RIPA lysis buffer (50 mM Tris-HCl, pH 7.4, 150 mM NaCl, 1% NP-40, 1 mM EDTA, 0.25% sodium deoxycholate, 1 mM NaF, 10 μM Na_3_VO_4_, 1 mM phenyl-methyl-sulfonyl-fluoride, 10 μg/mL aprotinin, 10 μg/mL pepstatin, and 10 μg/mL leupeptin). Bio-Rad protein assay was used to determine proteins concentration (BioRAD, Hercules, CA, USA). Western blot analysis on whole lysates was performed as previously described (PMID: 36168728). Anti-GAPDH (SC-47724) antibody was purchased from Santa Cruz Biotechnology (Irvine, CA, USA). Anti-phospho-cPLA2 S505, anti-phospho-5-LOX S271, and anti-phospho-5-LOX S663 were from Cell Signalling Technology (Denvers, MA, USA). Goat-anti-mouse (bs-0296G-HRP) and goat-anti-rabbit (bs-0295G-HRP) were from Bioss Antibodies (Woburn, MA, USA). Proteins were visualized by enhanced chemiluminescence reagent (Amersham Biosciences, Little Chalfont, Buckinghamshire, UK) and were quantified using densitometry (Chemidoc, BioRAD). Each experiment with the relative densitometric quantification was separately repeated at least three times.

### 2.5. Statistical Analysis

All data reported are expressed as means ± standard error mean (SEM) and are representative of at least three or more independent experiments. Statistical analyses were performed with unpaired *t*-test to compare the mean of two independent groups of experiments or by one-way analysis of variance (ANOVA). GraphPad Prism 7 (GraphPad Software Inc., San Diego, CA, USA) was used to compare more than two experiments. A *p* value of less than 0.05 was considered to be statistically significant.

## 3. Results and Discussion

### 3.1. FPR2 Stimulation Induces Arachidonic Acid Release by Activating cPLA2

We previously demonstrated that FPR2 stimulation triggers the metabolic reprogramming of CaLu-6 cells by modulating aerobic glycolysis, PPP, glutamine transport, and the de novo synthesis of pyrimidine nucleotides [44,45,46]. Besides glycolysis and glutamine metabolism, an emerging role of lipids in metabolic reprogramming of many types of cancers cells has been observed [48].

**Figure 1:**
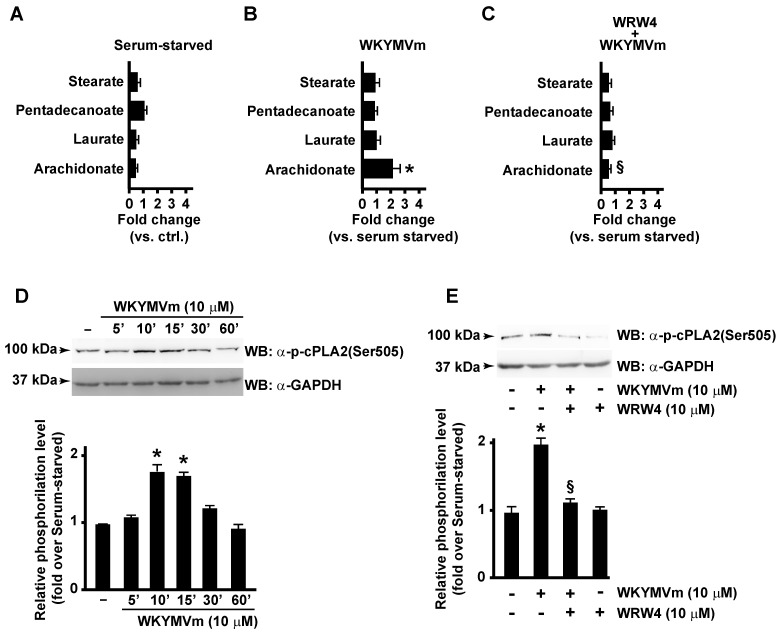
FPR2 promotes arachidonic acid metabolism by cPLA2 Serine 505 phosphorylation. FPR2 stimulation induces arachidonic acid metabolism in CaLu-6 cells (**A**–**C**). Bar graphs represent the metabolomic analysis of stearate, pentadecanoate, laurate, and arachidonate levels detected in 24 h serum-starved CaLu-6 cells (**A**), 10 μM WKYMVm-stimulated (WKYMVm) (**B**) and in 10 μM WRWWWW (WRW4)-pre-treated and WKYMVm-stimulated (WKYMVm + WRW4) (**C**) CaLu-6 cells. Growing cells (ctrl) were serum-starved for 24 h and then stimulated or not with 10 μM WKYMVm for 1 h in presence or absence of 10 μM WRW4. Metabolomic analysis was performed as described in Materials and Methods. cPLA2 Serine 505 (Ser505) phosphorylation is promoted by FPR2 stimulation with WKYMVm (**D**,**E**). Serum-starved CaLu-6 cells were exposed to 10 μM WKYMVm for 5, 10, 30, or 60 min (**D**), or pre-treated for 15 min with WRW4 before the stimulation with WKYMVm (**E**). Sixty micrograms of whole lysates were resolved on 10% SDS-PAGE and incubated with an anti-phospho-cPLA2 (Ser505) (α-p-cPLA2(Ser505)) antibody. An anti-GAPDH (α-GAPDH) antibody was used as control for protein loading. Data are representative of three independent experiments. * *p* < 0.05 compared to unstimulated cells. § *p* < 0.05 compared to stimulated cells.

By metabolomic analysis, we observed that AA concentration was significantly enhanced in WKYMVm-stimulated CaLu-6 cells (Figure 1B) compared to unstimulated cells (Figure 1A), whereas concentrations of other fatty acids, such as stearate, pentadecanoate, and laurate, were unaffected (Figure 1A,B). AA levels remained unchanged when cells were preincubated with the FPR2 antagonist WRWWWW (WRW4) (Figure 1C), thus indicating that it depends on FPR2 stimulation.

Three main mechanisms allow cells to obtain AA: (i) direct exogenous uptake via specific transporters, such as CD36, and TWIK-related AA-stimulated K+ (TRAAK) channels; (ii) de novo synthesis from linoleic acid; (iii) cleavage of the sn-2 position of existing membrane phospholipids through the enzymatic activity of cPLA2 [49,50,51].

cPLA2 activity is tightly regulated in cells by at least three mechanisms. The increase in the intracellular Ca^2+^ concentration represents a key regulator of cPLA2 activity in several cell types [52,53]. In addition to Ca^2+^, cPLA2 is also regulated by intracellular lipids, which allosterically activate the enzyme and increase its residence time in membranes [54]. cPLA2 is also regulated by phosphorylation at Ser505, Ser515, and Ser727 residues, which controls agonist-induced AA mobilization [55]. Ser515 and Ser727 phosphorylation depends on cell type and stimulation conditions, whereas only Ser505 phosphorylation is required for cPLA2 full activation and cell membrane translocation [55].

In WKYMVm-stimulated cells, we observed a time-dependent increase in cPLA2 Ser505 phosphorylation (Figure 1D), which was prevented by preincubation with the FPR2 antagonist (Figure 1E).

FPR2 ability to induce an increase in PLA2 expression was previously observed in SAA-stimulated cells [56], as well as in conjunctival goblet cells and in neutrophils incubated with ANXA1 or WKYMVm, respectively [57,58].

Our results demonstrate, for the first time, that FPR2 stimulation triggers cPLA2 phosphorylation at Ser505 residue, thereby suggesting that cPLA2 activation can contribute to the concomitant increase in AA concentration.

### 3.2. FPR2-Mediated cPLA2 Ser505 Phosphorylation Depends on NOX Activation

Ser505 residue of cPLA2 is phosphorylated by different kinases, such as ERKs [11] and p38MAPK [12]. Furthermore, there is increasing evidence that NOX-dependent ROS generation can activate cPLA2 [59] and that hydrolytic products of cPLA2, including AA, could enhance NOX activity [60,61,62]. The assembled NOX complex serves as a target for anchoring cPLA2 to the plasma membranes [63]. Therefore, it is conceivable that these two enzymes share a common mechanism for activation by intracellular kinases. ERKs and p38MAPK trigger both cPLA2 phosphorylation and ROS production, and both events are prevented by NADPH oxidase inhibitors [64].

Several GPCR agonists elicit an increase in NOX-dependent ROS concentration and trigger the activity of several kinases, such as p38MAPK, ERKs, and JNK, which are able to phosphorylate cPLA2 [65,66]. These kinases can be also activated by the canonical pathway of TKRs elicited by GPCR-dependent TKRs transactivation [67].

Previously, we demonstrated that intracellular domains of activated FPR2 mediate signaling to G-proteins, which trigger several agonist-dependent signaling cascades, including activation of PLC, PKC isoforms, p38MAPK, PI3K/Akt, and MAPK pathway. Phosphorylation of cytosolic tyrosine kinases, TKRs transactivation, phosphorylation and nuclear translocation of regulatory transcriptional factors, and release of calcium and production of oxidants also belong to the distinct intracellular pathways elicited by FPR2 [32].

**Figure 2:**
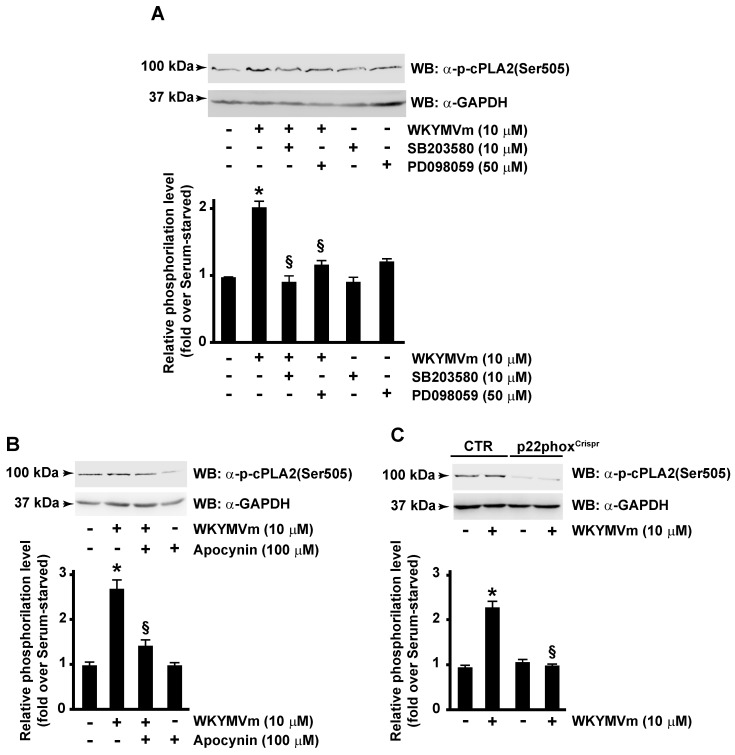
cPLA2 serine 505 phosphorylation requires both p38MAPK, ERKs and NADPH oxidase activation. FPR2-WKYMVm-mediated cPLA2 serine 505 phosphorylation depends on p38MAPK, ERKs (**A**), and NADPH oxidase activation (**B**,**C**). Serum-starved CaLu-6 cells were stimulated or not for 10 min with 10 μM WKYMVm in presence or absence of 10 μM SB203580 or 50 μM PD098059 (**A**), or 100 μM apocynin (**B**). CaLu-6-control^Crispr/Cas9^ cells (CTR) and p22phox^Crispr/Cas9^ (p22phox^Crispr^) cells were serum starved for 24 h and then stimulated with WKYMVm (**C**). Sixty micrograms of whole lysates was resolved on 10% SDS-PAGE and incubated with an anti-phospho-cPLA2 (Ser505) (α-p-cPLA2(Ser505)) antibody. An anti-GAPDH (α-GAPDH) antibody was used as control for protein loading. Data are representative of four independent experiments. * *p* < 0.05 compared to unstimulated cells. § *p* < 0.05 compared to stimulated cells.

Therefore, we analyzed molecular mechanisms involved in FPR2-induced cPLA2 phosphorylation by pretreating cells with selective inhibitors of MEK and p38MAPK. Cells were preincubated with PD098059 or SB203580 before WKYMVm stimulation, and in Western blot experiments, we observed that both inhibitors prevent cPLA2 phosphorylation at Ser505 residue (Figure 2A). Previously, we demonstrated that, in CaLu-6 cells, WKYMVm stimulation induces ERK-dependent p47^phox^ phosphorylation and membrane translocation and, in turn, NOX-dependent superoxide generation [38]. Therefore, we investigated the possible involvement of NOX in cPLA2 Ser505 phosphorylation by treating cells with apocynin, a selective inhibitor of NOX activity, before agonist stimulation. Obtained results demonstrated that specific block of NOX function prevented cPLA2 Ser505 phosphorylation (Figure 2B). Consistently, cPLA2 Ser505 phosphorylation was not observed following FPR2 stimulation with WKYMVm of the p22^phox^CaLu-6^Crispr/Cas9^ cells, which lack the p22^phox^ gene encoding a critical component of the superoxide-generating NOX [40] (Figure 2C). Taken together, these results prove that FPR2 modulates cPLA2 activation in an ERK-, p38MAPK-, and NOX-dependent manner.

Notably, ROS play an important role in inflammation processes and in signal transduction. According with our findings, NOX activation increases ROS levels that in turn inactivate the function of phosphothyrosine and serine/threonine phosphatases containing cysteine moieties within their active centers, which are susceptible to oxidation by ROS. Therefore, inactivation of phosphatases results in activation of MAPKs, p38MAPK, and ERKs which, in turn, activate cPLA2 [68]. Very interestingly, protein phosphatase inhibition also induces activation of non-receptor tyrosine kinase family Src, which, in turn, leads to EGFR phosphorylation [38] and thus to the transduction of this signal into MAPK cascades [69], further improving cPLA2 activation.

### 3.3. FPR2 Stimulation Induces 5-LOX Activation

Eicosanoids, including PGs, TXs, LTs, and lipoxins, are generated during the various phases of AA metabolism along the COX and LOX pathways [70]. Release of AA and activation of 5-LOX, in response to several stimuli, initiates the biosynthesis of proinflammatory LTs, a family of lipid mediators with pivotal roles in inflammatory disorders [71].

In addition to the normal expression in the various leukocyte types, aberrant expression of 5-LOX has been detected in many tumor cells [72,73,74] and, besides inflammatory processes, 5-LOX is involved in cell differentiation, oxidative stress, and in the progression of different diseases [75,76,77].

In the resting cell, 5-LOX is localized in either the cytosol or a soluble compartment inside the nucleus. Activation of cellular 5-LOX involves enzyme translocation to the nuclear envelope, where it colocalizes with cPLA2 and FLAP [78,79,80].

5-LOX is a substrate for several protein kinases, and phosphorylation of different residues has divergent consequences for 5-LOX subcellular localization and activity. Phosphorylation at Ser271 and Ser663 residues increases 5-LOX expression and is strongly promoted by unsaturated fatty acids, including AA. Enhanced 5-LOX activity following dual phosphorylation at Ser271 and Ser663 residues is observed when intracellular Ca^2+^ levels are low and, thus, insufficient to activate 5-LOX alone. In addition, Ser271 phosphorylation promotes nuclear localization of 5-LOX [81]. An increase in cAMP levels activates PKA, which represses 5-LOX activity through phosphorylation at Ser523 residue [82]. This phosphorylation prevents nuclear import of the enzyme and, in turn, its cytoplasmic enrichment [83].

**Figure 3:**
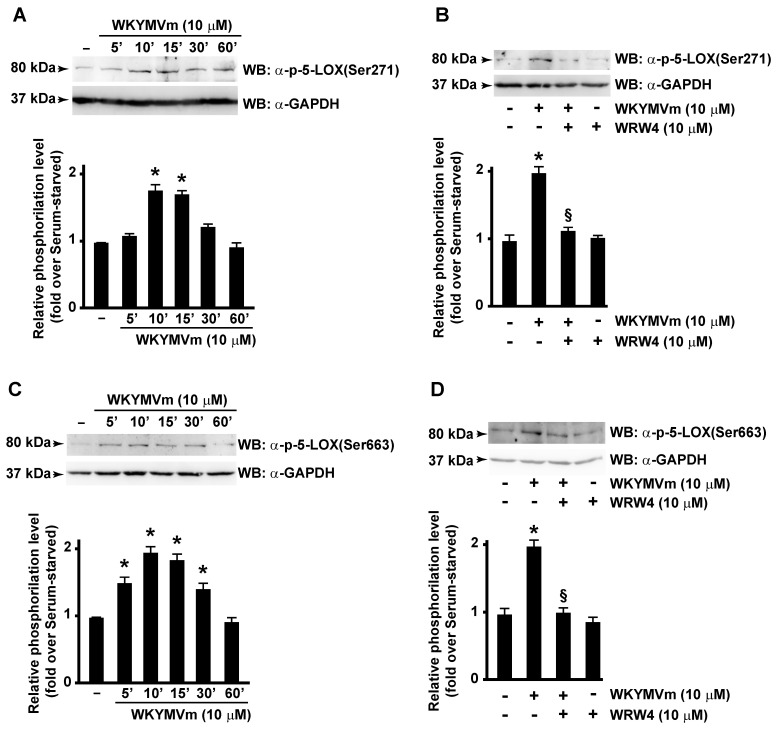
FPR2 stimulation induces 5-LOX activation by enhancing Serine 271 and Serine 663 phosphorylation. FPR2 exposure to WKYMVm enhances 5-LOX phosphorylation on Serine 271 and Serine 663. Serum-starved CaLu-6 cells were stimulated with WKYMVm for 5, 10, 15, 30, and 60 min (**A**,**C**) in presence or absence of WRW4 (**B**,**D**). Fifty micrograms of whole lysates was resolved on 10% SDS-PAGE and incubated with an anti phospho-5-LOX (Ser271) (α-p-5-LOX(Ser271)) or anti phospho-5-LOX (Ser663) (α-p-5-LOX(Ser663)) antibodies. An anti-GAPDH (α-GAPDH) antibody was used as control for protein loading. Data are representative of three independent experiments. * *p* < 0.05 compared to unstimulated cells. § *p* < 0.05 compared to stimulated cells.

In this scenario, we investigated the ability of FPR2 stimulation to trigger 5-LOX activation. By analyzing WKYMVm-treated CaLu-6 cells, we demonstrated that FPR2 stimulation induces Ser271 (Figure 3A) and Ser663 (Figure 3C), but not Ser523, phosphorylation of 5-LOX, whereas preincubation with WRW4, a selective FPR2 antagonist, prevented both Ser271 (Figure 3A) and Ser663 phosphorylation (Figure 3B,D). These findings, for the first time, demonstrate that 5-LOX is specifically activated by FPR2 stimulation.

### 3.4. p38MAPK, ERKs and NOX Are Required for FPR2-Dependent 5-LOX Phosphorylation

p38MAPK-regulated MAPK-activated protein kinases (MAPKAPKs) have been identified as the kinases that phosphorylate 5-LOX at Ser271 residue in vitro [84,85].

In PMN, AA and fMLP, an FPR1 agonist, activate p38MAPK, leading to 5-LOX activation [84]. ERKs have been described as kinases that phosphorylate 5-LOX at Ser663 residue, and dual phosphorylation by ERK2 and p38MAPKAPKs at different sites is necessary for AA-induced 5-LOX activation [86]. AA plays a central role in the convergence of MAPK signaling cascades, leading to phosphorylation and activation of 5-LOX. Synergistic actions of MAPK pathways were also observed for the activation of cPLA2 [13,87].

**Figure 4:**
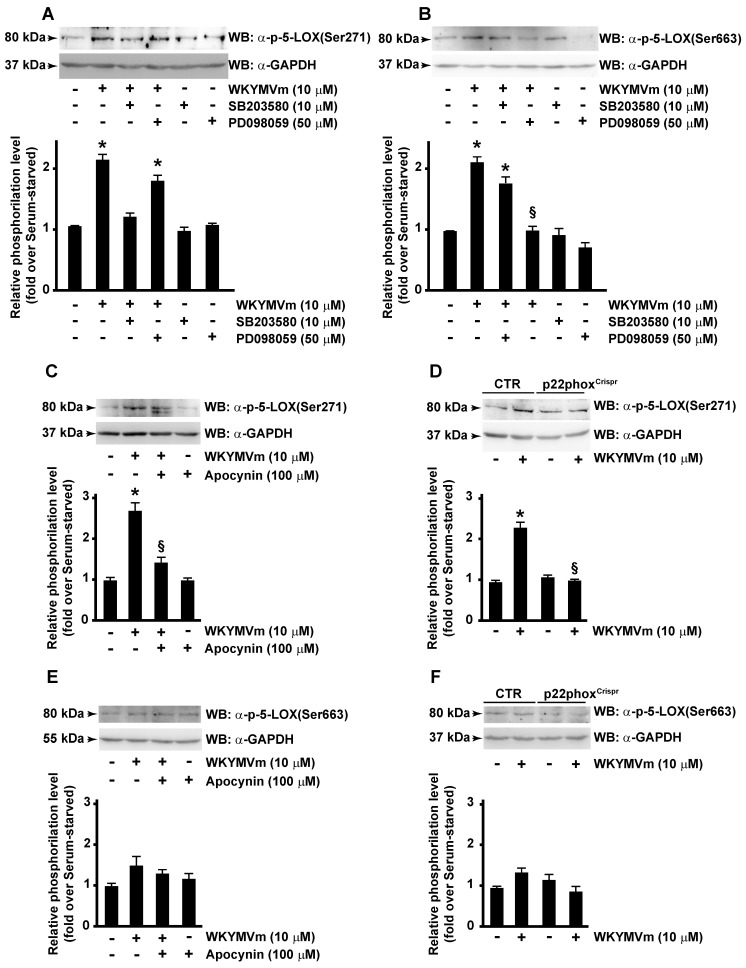
FPR2-mediated 5-LOX phosphorylation depends on p38MAPK, ERKs and NADPH oxidase activation. Serum-deprived CaLu-6 cells were pre-incubated with SB203580 or PD098059 (**A**,**B**) or apocynin (**C**,**E**) and stimulated or not with WKYMVm for 10 min. CaLu-6-control^Crispr/Cas9^ cells (CTR) and p22phox^Crispr/Cas9^ (p22phox^Crispr^) cells were serum-starved for 24 h and then stimulated with WKYMVm (**D**,**F**). Fifty micrograms of whole lysates was resolved on 10% SDS-PAGE and incubated with an anti phospho-5-LOX (Ser271) (α-p-5-LOX(Ser271)) or anti phospho-5-LOX (Ser663) (α-p-5-LOX(Ser663)) antibodies. An anti-GAPDH (α-GAPDH) antibody was used as control for protein loading. Data are representative of three independent experiments. * *p* < 0.05 compared to unstimulated cells. § *p* < 0.05 compared to stimulated cells.

ERKs are typically activated by growth-related stimuli through the Raf-1/MEK1/2 protein kinase cascade. However, upon activation, FPR2 triggers several agonist-dependent signal transduction cascades, including Ras-MAPK pathway, PLC, and p38MAPK activation [32]. Consistently, we observed that, upon WKYMVm stimulation, phosphorylation of 5-LOX at Ser271 residue was prevented in CaLu-6 cells pretreated with SB203580, an inhibitor of p38MAPK signaling pathway (Figure 4A); similarly, Ser663 phosphorylation failed when cells were preincubated with the MAPK inhibitor PD098059 (Figure 4B). These results prove that FPR2 stimulation induces 5-LOX phosphorylation through FPR2-dependent p38MAPK and ERKs activation.

Because catalysis by 5-LOX requires oxidation of Fe^2+^ to Fe^3+^ in the active site of the enzyme, cellular redox conditions represent a crucial factor that modulates 5-LOX activity. Conditions that promote the formation of ROS upregulate 5-LOX, whereas reducing conditions, as well as the presence of suitable thiols (GSH or DTT), prevent 5-LOX activity [81,88].

In mammalian cells, ROS are generated via a variety of cellular oxidative processes, including the activities of NOX, xanthine oxidases, and the mitochondrial respiratory chain. NOX-generated ROS are the best characterized examples of ROS, although they are also generated by the oxidative metabolism of AA, released from the membrane phospholipids via cPLA2 activity. In fact, LOX- and COX-generated AA metabolites can induce ROS generation by stimulating NOX, highlighting a potential signaling connection between LOX/COX metabolites and NOX [89].

We analyzed the role of NOX in FPR2-dependent phosphorylation of Ser271 and Ser663 of 5-LOX. CaLu-6 cells were stimulated with WYKMVm or preincubated with apocynin, a NOX inhibitor, before stimulation. Results showed that Ser271 phosphorylation of 5-LOX was prevented by apocynin (Figure 4C), whereas Ser663 phosphorylation was not affected by NOX-dependent modulations of redox state (Figure 4E). Accordingly, in p22^phox^Crispr/Cas9 cells, Ser271 phosphorylation (Figure 4D), but not Ser663 phosphorylation (Figure 4E), required functional expression of NOX.

Ser271 is phosphorylated by p38MAPK-regulated MAPKAPKs, and among MAPK families, p38MAPK is also activated when cells are exposed to various cellular stress. We previously proved that in CaLu-6 cells FPR2 stimulation with WKYMVm or ANXA1 induced NOX-dependent activation of p38MAPK [40]. Thus, an increase in ROS in these cells may activate p38MAPK and, consequently, 5-LOX by promoting Ser271 phosphorylation [90].

This latter evidence and the results herein presented strongly demonstrate that FPR2 signaling induces 5-LOX phosphorylation at Ser271 and Ser663, which requires p38MAPK and ERKs activation, respectively. Furthermore, since NOX inhibition prevents Ser271 phosphorylation, we can hypothesize that FPR2-dependent NOX activation might contribute to 5-LOX nuclear translocation.

## 4. Conclusions

In the last decade, much progress has been made in understanding the effects of ROS on inflammatory processes that are associated with many human diseases. In many cases, the disease, or its progression, is associated with NOX activation, which generates ROS that trigger inflammatory reactions, representing one of the risk factors for cancer.

**Figure 5:**
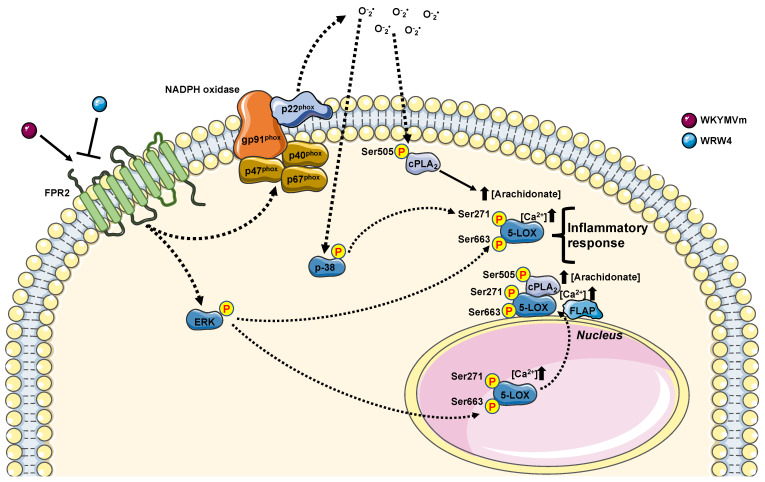
FPR2 stimulation contribute to regulate arachidonic acid metabolism. Upon WKYMVm incubation, FPR2 increases the concentration of arachidonate. FPR2, once stimulated, can contribute to arachidonic acid metabolism by phosphorylating cPLA2 on its Serine 505 (Ser505) in a p38MAPK-, ERKs-, and NADPH oxidase-dependent fashion. FPR2-cPLA2-mediated increase in arachidonic acid can improve 5-LOX activation by phosphorylating its Serine 271 (Ser271) and Serine 663 (Ser663). 5-LOX activation can be also promoted by an increase in intracellular Ca^2+^ concentration ([Ca^2+^]). Both 5-LOX phosphorylation required FPR2 stimulation. However, Ser271 required p38MAPK and NADPH oxidase activation, whereas Ser663 ERKs activation. 5-LOX, once phosphorylated, can migrate to the nuclear envelope, where it colocalizes with PLA2 and 5-lipoxygenase activating protein (FLAP), and in turn, it can contribute to generating anti-inflammatory mediators. Furthermore, 5-LOX phosphorylation at Ser271 promotes its nuclear localization, where it can favor biosynthesis of pro-inflammatory mediators.

Although 5-LOX catalyzes the first step of LTs molecules with proinflammatory functions, it is also involved in the biosynthesis of lipid mediators with anti-inflammatory properties, such as lipoxins. In many diseases, an imbalance between proinflammatory LTs and anti-inflammatory lipoxins is observed. However, some questions remain unresolved. How does intranuclear localization of 5-LOX confer a higher activity? Does 5-LOX have other roles inside the nucleus? Since inhibition of LTs biosynthesis shows beneficial effects in various inflammatory diseases, new findings on 5-LOX activation/translocation may lead to future novel therapeutic possibilities. In this context, the elucidation of the molecular mechanisms underlying inflammatory reactions triggered by NOX-dependent ROS generation (Figure 5) can provide new strategies to inhibit 5-LOX activation, whereas the control of FPR2-mediated nuclear import of 5-LOX might represent an important step in resolving the inflammatory process in many human diseases.

## Data Availability

The data presented in this study are available within the article. Other data that support the findings of this study are available upon request to the corresponding authors.
